# Successful Management of a 27-cm Mucinous Cystic Neoplasm of the Liver With Laparoscopic Excision: A Case Report

**DOI:** 10.7759/cureus.108687

**Published:** 2026-05-11

**Authors:** Medha Sravanthi Lomada, Ushast Dhir, Pallav Gupta, Ashish Kumar

**Affiliations:** 1 Gastroenterology and Hepatology, Sir Ganga Ram Hospital, New Delhi, IND; 2 Surgical Gastroenterology and Liver Transplantation, Sir Ganga Ram Hospital, New Delhi, IND; 3 Pathology, Sir Ganga Ram Hospital, New Delhi, IND

**Keywords:** biliary cystadenoma, giant liver cyst, hepatic cyst, laparoscopic liver surgery, mucinous cystic neoplasm, ovarian-type stroma

## Abstract

Mucinous cystic neoplasms of the liver (MCN-L) are rare cystic tumors defined by the presence of ovarian-type subepithelial stroma beneath mucin-secreting epithelium and carry a risk of malignant transformation, necessitating complete surgical excision. Giant MCN-L lesions are exceptionally uncommon, and data regarding minimally invasive management of such large tumors remain limited.

A 44-year-old woman (American Society of Anesthesiologists I) presented with a six-month history of progressive right-sided abdominal fullness. Contrast-enhanced computed tomography revealed a large 27 × 18 × 19 cm thin-walled, unilocular cystic lesion arising from the posterior segments of the right hepatic lobe, extending into the pelvis. Laboratory investigations demonstrated microcytic hypochromic anemia (hemoglobin 9.6 g/dL), with preserved liver and renal function. Serum tumor markers showed carcinoembryonic antigen of 0.70 ng/mL, carbohydrate antigen 19-9 of 11.33 U/mL, cancer antigen-125 of 13.3 U/mL, and alpha-fetoprotein of 9.27 ng/mL. Hydatid serology was negative.

The patient underwent laparoscopic right posterior sectionectomy (segments VI and VII) with cholecystectomy. Approximately 4.5 L of clear fluid was aspirated in a controlled manner to decompress the cyst and improve operative exposure. Complete excision of the cyst-replaced posterior sector was achieved. The operative time was approximately 210 minutes with an estimated blood loss of 150 mL.

Histopathological examination confirmed mucinous cystic neoplasm with mucin-secreting epithelium (cytokeratin 7 positive, cytokeratin 20 negative) and characteristic ovarian-type stroma expressing estrogen and progesterone receptors, without dysplasia or malignancy. The postoperative course was uneventful, and the patient was discharged in stable condition. At one-year follow-up, there was no evidence of recurrence.

This case highlights that laparoscopic right posterior sectionectomy is feasible for giant MCN-L, even when the lesion exceeds 25 cm, provided careful decompression and appropriate surgical expertise are available. At 27 cm, this represents one of the largest MCN-L successfully managed using a purely laparoscopic approach. Complete excision remains essential due to the malignant potential of these lesions.

## Introduction

Mucinous cystic neoplasms of the liver (MCN-L), previously termed biliary cystadenomas, are rare cystic tumors accounting for less than 5% of hepatic cysts [1]. They are defined by mucin-producing biliary epithelium with underlying ovarian-type stroma, typically without communication with the biliary tree [2]. This ovarian-type stroma is the key histopathologic feature that distinguishes MCN-L from other hepatic cystic lesions such as simple cysts, hydatid cysts, and intraductal papillary neoplasms of the bile duct [1,3].

MCN-L occur predominantly in middle-aged women and often present with nonspecific symptoms such as abdominal pain or fullness, although some are detected incidentally [1,4]. These lesions are clinically important due to their malignant potential, with reported rates of invasive carcinoma ranging from less than 5% to approximately 9% [1,3,4]. As preoperative imaging and biopsy cannot reliably exclude malignancy, complete surgical excision is recommended [1,5].

Most MCN-L are large at presentation, with a mean size of approximately 11 cm [1,6]. However, giant lesions exceeding 20 cm are uncommon, and experience with minimally invasive management of such cases remains limited. Nevertheless, advances in laparoscopic liver surgery have enabled minimally invasive resection in selected cases [7,8]. We report a case of a giant MCN-L measuring 27 cm, successfully managed with laparoscopic right posterior sectionectomy.

## Case presentation

A 44-year-old woman presented with a six-month history of progressive right-sided abdominal fullness and mild intermittent discomfort. She denied fever, jaundice, nausea, vomiting, weight loss, or changes in bowel habits. Her medical history was unremarkable, with no prior abdominal surgeries or significant comorbidities. There was no history of oral contraceptive or hormonal therapy use, alcohol consumption, smoking, or family history of hepatobiliary neoplasms. Her American Society of Anesthesiologists physical status was class I.

On examination, the abdomen was soft and nontender with visible distension. No discrete mass was palpable, and there was no hepatosplenomegaly. Vital signs were within normal limits. Laboratory investigations demonstrated microcytic hypochromic iron-deficiency anemia. Liver and renal function tests and coagulation parameters were within normal limits. Serum tumor markers showed normal levels of carcinoembryonic antigen (CEA) (0.70 ng/mL), carbohydrate antigen (CA) 19-9 (11.33 U/mL), and cancer antigen (CA)-125 (13.3 U/mL), with alpha-fetoprotein (AFP) at 9.27 ng/mL. Echinococcal serology (IgG enzyme-linked immunosorbent assay) was negative. Although AFP was minimally above the stated laboratory reference range, there was no radiologic or histopathologic evidence of malignancy.

Abdominal ultrasonography demonstrated a large anechoic cystic lesion measuring 21 × 21 × 19 cm arising from the right hepatic lobe, displacing the liver and gallbladder to the left. The lesion appeared unilocular with thin walls and no internal septations or solid components.

**Figure 1:**
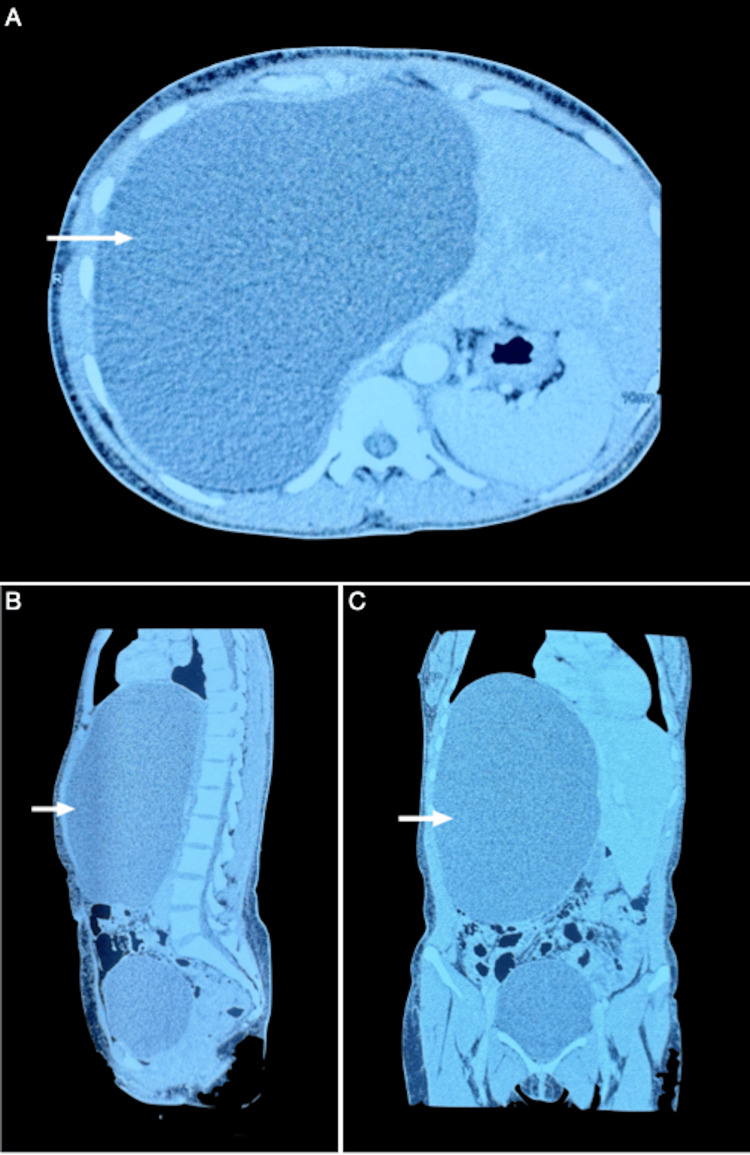
Contrast-enhanced CT of the abdomen demonstrating a 27 × 18 × 19 cm thin-walled, unilocular cystic lesion arising from the posterior segments (VI and VII) of the right hepatic lobe. The lesion extends inferiorly into the pelvis, causing significant mass effect on the right kidney, inferior vena cava, pancreas, and bowel loops. No mural nodules, septations, or calcifications are identified. The remaining liver parenchyma is unremarkable. (A) Axial, (B) sagittal, and (C) coronal views

Contrast-enhanced computed tomography of the abdomen and pelvis revealed a 27 × 18 × 19 cm thin-walled, fluid-attenuation cyst arising from segments VI and VII of the right hepatic lobe (Figure 1). The lesion extended into the pelvis, causing significant mass effect on the right kidney, inferior vena cava, pancreas, and bowel loops. Fat planes were preserved, with no evidence of invasion. No mural nodules, septations, or calcifications were identified, and the remaining liver parenchyma was unremarkable.

The discrepancy in lesion dimensions between ultrasonography (21 × 21 × 19 cm) and CT (27 × 18 × 19 cm) is likely attributable to differences in imaging modality and measurement planes. Ultrasonography is operator-dependent and may be limited in capturing the maximal dimension in large lesions, whereas CT provides standardized cross-sectional imaging, allowing more precise assessment of lesion extent along different axes. Variations in patient positioning and measurement orientation may also contribute to these differences.

Magnetic resonance imaging with magnetic resonance cholangiopancreatography confirmed a cystic lesion with homogeneous T2 hyperintensity and T1 hypointensity, consistent with simple fluid content. There was no communication with the biliary tree, and only minimal wall enhancement was noted. Although the lesion appeared unilocular without septations or nodularity, this does not exclude MCN-L [6,9].

Based on imaging, the differential diagnosis included a simple hepatic cyst, mucinous cystic neoplasm, and hydatid cyst. Given the large size, progressive symptoms, and inability to exclude a neoplastic process, surgical excision was planned.

The patient underwent laparoscopic right posterior sectionectomy (segments VI and VII) with concurrent cholecystectomy under general anesthesia. A five-port technique was used with the patient in the supine position with slight left lateral tilt. Three-dimensional visualization and indocyanine green (ICG) fluorescence guidance were used to facilitate dissection and assess for biliary communication [10].

Preoperative laboratory investigations are summarized in Table 1. Findings were notable for microcytic, hypochromic iron-deficiency anemia. Liver and renal function tests and coagulation parameters were largely within normal limits. Serum tumor markers showed normal CEA (0.70 ng/mL), CA 19-9 (11.33 U/mL), and CA-125 (13.3 U/mL) levels, with an AFP of 9.27 ng/mL. Hydatid serology was negative.

**Table 1:** Preoperative laboratory investigations AFP: alpha-fetoprotein; CA 19-9: carbohydrate antigen 19-9; CA-125: cancer antigen 125; CEA: carcinoembryonic antigen; AST: aspartate aminotransferase; SGOT: serum glutamic-oxaloacetic transaminase; ALT: alanine aminotransferase; SGPT: serum glutamic-pyruvic transaminase; PT: prothrombin time; INR: international normalized ratio; aPTT: activated partial thromboplastin time

Parameter	Result	Reference range
Hemoglobin	9.6 g/dL	12.0-15.0 g/dL
Total leukocyte count	4,900/cumm	4,000-10,000/cumm
Platelet count	1.58 lakh/cumm	1.5-4.5 lakh/cumm
AST (SGOT)	25 U/L	17-59 U/L
ALT (SGPT)	19 U/L	21-72 U/L
Total bilirubin	1.24 mg/dL	0.3-1.2 mg/dL
Albumin	4.8 g/dL	3.5-5.0 g/dL
Creatinine	0.68 mg/dL	0.6-1.1 mg/dL
PT	12.8 seconds	11.5-14.1 seconds
INR	1.00	0.75-1.20
aPTT	33.5 seconds	27.2-34.5 seconds
CEA	0.70 ng/mL	<5.0 ng/mL
AFP	9.27 ng/mL	<8.1 ng/mL
CA 19-9	11.33 U/mL	<35 U/mL
CA-125	13.3 U/mL	<35 U/mL
Hydatid serology	0.20 OD	Negative

**Figure 2:**
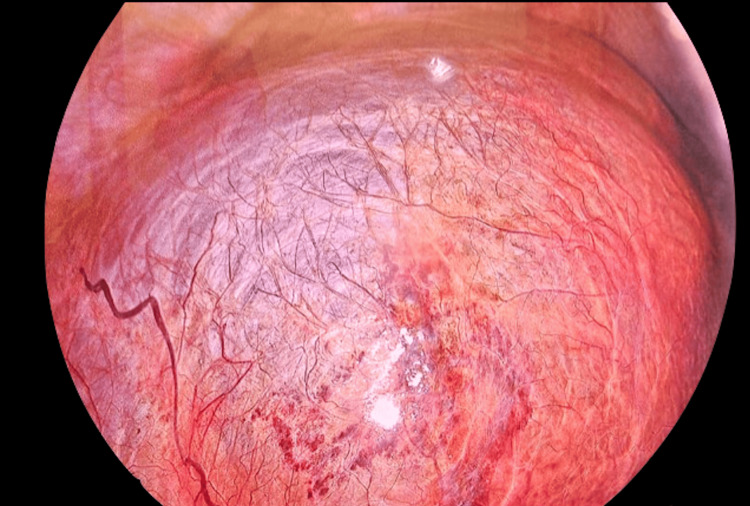
Intraoperative laparoscopic view demonstrating a large, tense hepatic cystic lesion with a thin, translucent wall occupying a substantial portion of the abdominal cavity prior to decompression. The cyst wall appears smooth without visible solid components or nodularity. The massive size of the lesion limited working space and visualization, necessitating controlled aspiration as an initial step

Intraoperatively, a large cystic lesion arising from the right posterior segments was identified, occupying a substantial portion of the abdominal cavity and extending into the pelvis. The cyst had replaced the entire posterior sector, and the wall appeared thin and translucent without solid components or nodularity (Figure 2).

Controlled cyst decompression was performed as an initial step to restore adequate working space. Approximately 4.5 L of clear fluid was aspirated using a closed-suction system to minimize spillage, and a sample was sent for cytological analysis. The abdominal cavity was irrigated following aspiration.

Following decompression, cholecystectomy was performed due to the proximity of the gallbladder to the lesion. Intraoperative ultrasound was used to delineate the relationship of the cyst to vascular structures. As the cyst had replaced the entire posterior sector, a formal right posterior sectionectomy was performed. The right posterior pedicle was identified and ligated, followed by parenchymal transection along the intersegmental plane using energy devices, with careful control of intrahepatic vessels and bile ducts.

The resection surface was inspected for bile leaks using ICG fluorescence, and none were identified. Hemostasis was achieved, and the specimen was retrieved in an endobag. A drain was placed in the subhepatic space. The procedure was completed without complications, with an operative time of approximately 210 minutes and an estimated blood loss of 150 mL. No blood transfusion was required.

**Figure 3:**
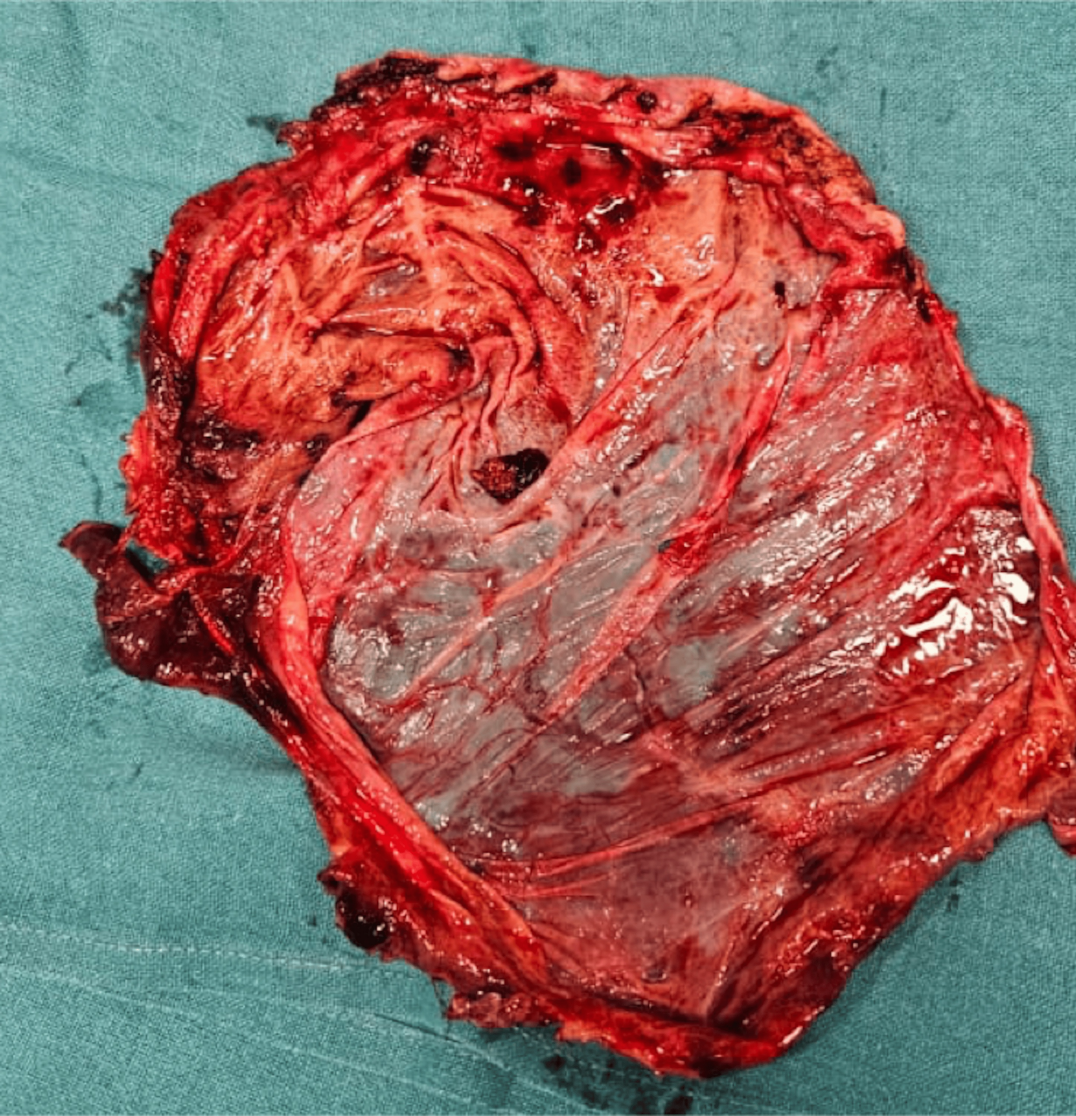
Gross specimen of excised hepatic cyst wall and resected liver parenchyma following right posterior sectionectomy. The cyst wall is thin (up to 0.2 cm in thickness) and translucent, with a smooth, glistening inner surface. No papillary projections, solid areas, or mural nodules are identified on gross examination. The specimen demonstrates complete excision achieved through formal anatomical resection

Gross examination revealed a large cystic specimen with a thin, smooth wall measuring up to 0.2 cm in thickness (Figure 3). The inner surface was smooth and glistening, without papillary projections or solid areas.

**Figure 4:**
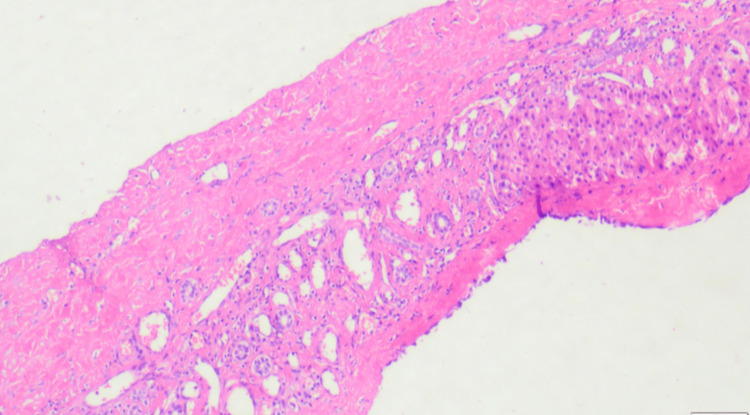
Histopathological features of mucinous cystic neoplasm of the liver Histopathological examination (hematoxylin and eosin stain, ×200 magnification) demonstrating a cyst lined by a single layer of columnar to cuboidal mucin-secreting epithelium resembling biliary epithelium. Beneath the epithelial layer, dense ovarian-type stroma composed of spindle cells with elongated nuclei is present. Immunohistochemistry confirmed estrogen and progesterone receptor positivity in the stromal cells (not shown). No dysplasia, atypia, or invasive carcinoma is identified

Microscopic examination showed a cyst lined by a single layer of columnar to cuboidal mucin-secreting epithelium resembling biliary epithelium (Figure 4). Beneath the epithelial lining, dense ovarian-type stroma composed of spindle cells with elongated nuclei was identified. Immunohistochemistry demonstrated positivity for estrogen and progesterone receptors in stromal cells. The epithelial cells were positive for cytokeratin 7 and negative for cytokeratin 20, consistent with a biliary phenotype.

No dysplasia, atypia, or invasive carcinoma was identified. Cytological examination of the cyst fluid showed benign epithelial cells without malignant features. These findings were consistent with a noninvasive MCN of the liver.

The postoperative course was uneventful. Drain output was minimal and serous, with no evidence of bile leak, and the drain was removed in the early postoperative period. The patient tolerated oral intake well and achieved adequate pain control with oral analgesics. Liver function tests remained stable, and she was discharged in stable condition.

At follow-up, the patient reported complete resolution of abdominal fullness and discomfort and returned to normal daily activities within two weeks. Ultrasonography at three and twelve months showed no evidence of recurrence or residual fluid collection. At 12 months postoperatively, the patient remained asymptomatic with normal liver function tests and no radiological evidence of recurrence. Long-term surveillance with periodic imaging is planned (Table 2).

**Table 2:** Clinical timeline of patient presentation, diagnosis, treatment, and follow-up

Date/timepoint	Event
June 2023	Onset of progressive right-sided abdominal fullness
November 27, 2023	Abdominal ultrasonography demonstrated a 21 × 21 × 19 cm cystic lesion arising from the right hepatic lobe
November 30, 2023	Contrast-enhanced CT abdomen demonstrated a 27 × 18 × 19 cm cystic lesion involving segments VI and VII of the liver
December 2023	MRI with MRCP confirmed the absence of biliary communication
December 11, 2023	Preoperative laboratory investigations and tumor marker assessment were performed
December 14, 2023	Laparoscopic right posterior sectionectomy (segments VI and VII) with cholecystectomy performed
Postoperative period	Uneventful recovery without major complications
3-month follow-up	No recurrence on ultrasonography
12-month follow-up	Asymptomatic with no radiological evidence of recurrence

Written informed consent was obtained from the patient for publication of this case report and accompanying images.

## Discussion

This case is notable for the large size of the lesion (27 cm), the successful use of a purely laparoscopic approach for complete excision, and the diagnostic uncertainty on preoperative imaging. Most MCN-L are smaller, with a reported mean size of approximately 11 cm [1,6]. Giant lesions exceeding 20 cm are uncommon, with most reported series describing smaller lesion sizes [1,6]. At 27 cm, this represents one of the largest MCN-L reported and among the largest managed laparoscopically.

The primary technical challenge in managing giant hepatic cystic lesions laparoscopically is limited working space and visualization. In our patient, controlled cyst decompression was a key step, allowing reduction in size and facilitating safe dissection. This approach has been described in the management of large hepatic cysts and can enable minimally invasive resection in selected cases [7,11]. However, aspiration must be followed by complete excision, as incomplete procedures such as fenestration or deroofing are associated with recurrence [6,12].

Although cyst decompression is effective, it carries a theoretical risk of spillage and peritoneal dissemination in mucinous neoplasms. To minimize this risk, we employed a closed suction technique with controlled aspiration under direct visualization, followed by irrigation of the operative field. Current evidence suggests that the risk of dissemination in benign MCN-L is low, but careful technique remains essential [1,3,4].

The choice of surgical approach in MCN-L depends on lesion characteristics. While enucleation may be appropriate for peripherally located lesions, formal hepatic resection is preferred when there is no clear plane between the cyst and surrounding parenchyma [1,4]. In our case, the cyst had replaced the entire posterior sector, necessitating a posterior sectionectomy to ensure complete excision.

Preoperative diagnosis of MCN-L remains challenging. Although multilocular morphology and left-lobe location are more typical, unilocular lesions and right-lobe involvement have been reported and do not exclude the diagnosis [7,9]. The absence of classical imaging features in this case contributed to diagnostic uncertainty and highlights the limitations of imaging alone. Definitive diagnosis relies on histopathological identification of ovarian-type stroma. The mild isolated AFP elevation in this case was considered nonspecific in the absence of radiologic or histopathologic evidence of malignancy.

In endemic regions, hydatid cyst remains an important differential diagnosis. In our patient, negative serology and absence of characteristic imaging features helped exclude this possibility preoperatively.

This report has limitations inherent to a single case, including limited follow-up duration and the absence of cyst fluid tumor marker analysis. Additionally, the procedure was performed in a specialized center with advanced laparoscopic expertise, which may limit generalizability.

Overall, laparoscopic resection of giant MCN-L is feasible in selected patients, provided careful patient selection, controlled decompression, and complete excision are ensured.

## Conclusions

Giant MCN-L, although rare, can be safely managed laparoscopically even when exceeding 25 cm, provided adequate decompression and appropriate surgical expertise are available. Complete excision remains essential due to the malignant potential of these lesions.

This case highlights the importance of considering mucinous cystic neoplasm in the differential diagnosis of large hepatic cystic lesions, even when imaging features are atypical, and supports the use of echinococcal serology in endemic regions.
